# Solid-Phase Microextraction-Aided Capillary Zone Electrophoresis-Mass Spectrometry: Toward Bottom-Up Proteomics of Single Human Cells

**DOI:** 10.1021/jasms.3c00429

**Published:** 2024-03-21

**Authors:** Jorge A. Colón Rosado, Liangliang Sun

**Affiliations:** Department of Chemistry, Michigan State University, 578 S Shaw Lane, East Lansing, Michigan 48824, United States

**Keywords:** CZE-MS, solid-phase microextraction, single-cell proteomics, bottom-up proteomics, human cells

## Abstract

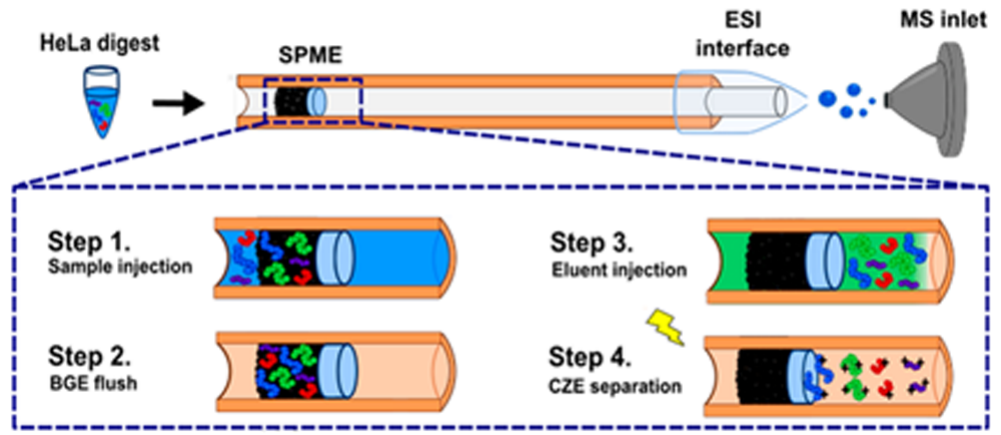

Capillary zone electrophoresis-mass spectrometry (CZE-MS) has been recognized as a valuable technique for the proteomics of mass-limited biological samples (i.e., single cells). However, its broad adoption for single cell proteomics (SCP) of human cells has been impeded by the low sample loading capacity of CZE, only allowing us to use less than 5% of the available peptide material for each measurement. Here we present a reversed-phase-based solid-phase microextraction (RP-SPME)-CZE-MS platform to solve the issue, paving the way for SCP of human cells using CZE-MS. The RP-SPME-CZE system was constructed in one fused silica capillary with zero dead volume for connection via in situ synthesis of a frit, followed by packing C8 beads into the capillary to form a roughly 2 mm long SPME section. Peptides captured by SPME were eluted with a buffer containing 30% (v/v) acetonitrile and 50 mM ammonium acetate (pH 6.5), followed by dynamic pH junction-based CZE-MS. The SPME-CZE-MS enabled the injection of nearly 40% of the available peptide sample for each measurement. The system identified 257 ± 24 proteins and 523 ± 69 peptides (*N* = 2) using a Q-Exactive HF mass spectrometer when only 0.25 ng of a commercial HeLa cell digest was available in the sample vial and 0.1 ng of the sample was injected. The amount of available peptide is equivalent to the protein mass of one HeLa cell. The data indicate that SPME-CZE-MS is ready for SCP of human cells.

## Introduction

The field of single-cell proteomics (SCP) has advanced significantly in recent years regarding the proteome coverage of single cells due to the improvement of sample preparation, peptide separations, and mass spectrometry (MS).^1−15^ Shifting from traditional bulk analysis to exploring the proteome at single cell levels offers valuable biological insights into the roles played by cell-to-cell heterogeneity in disease and development.^16−18^

Bottom-up proteomics (BUP) is a widely used strategy in SCP.^7,11−13^ BUP is preferred in SCP because of its superior sensitivity compared to top-down proteomics (TDP),^19−21^ which studies intact proteoforms instead of peptides. Reversed-phase liquid chromatography (RPLC)-tandem mass spectrometry (MS/MS) has been established as a powerful and commonly used technique for SCP.^16,22,23^ Capillary zone electrophoresis (CZE)-MS/MS has also been suggested as a powerful tool for SCP due to its high efficiency and high sensitivity for peptide separation and detection.^12,13,16,24−26^ CZE separates analytes according to their electrophoretic mobilities, which correspond to their charge-to-size ratios, in an open tubular capillary under an electric field. CZE does not have a stationary phase during the separation, and samples are injected into the separation capillary directly from sample vials via applying a pressure or a voltage without the need of sample loop and transfer tubing. It has been well documented that CZE-MS outperforms RPLC-MS in terms of peptide and protein identifications (IDs) for mass-limited biological samples.^13,16,27−29^ CZE-MS offers an extremely high sensitivity for peptide detection. For example, it has been reported that CZE-MS can detect 1 zmol (∼600 molecules) of angiotensin using a Q-Exactive HF mass spectrometer.^30^ Nearly 100 proteins were identified from single neurons using CZE-MS/MS.^31^ Over 700 proteins were identified by CZE-MS/MS when a subng amount of HeLa cell lysate digest was loaded into the capillary.^32^ About 60 proteins were identified by CZE-MS/MS when only 400 fg of an *E. coli* proteome digest was loaded for the measurement.^33^ However, the wide adoption of CZE-MS/MS in SCP of human cells has been impeded by its low sample loading capacity (usually less than 1% of the total capillary volume, low nL), only allowing the use of typically less than 5% of the available peptide material for each CZE-MS measurement.^24,31−33^

To address this issue, different sample stacking techniques have been investigated to increase sample loading capacity of CZE for CE-MS-based proteomics applications, i.e., dynamic pH junction,^34−38^ transient isotachophoresis,^13,39^ capillary isoelectric focusing,^40−44^ and solid-phase microextraction (SPME).^13,45−47^ SPME is particularly interesting because it can allow the use of all of the available peptide material for CZE-MS measurement. In a typical SPME-CZE-MS experiment, peptides in a sample vial can be fully loaded onto the SPME because they can be captured by the SPME through hydrophobic interaction (i.e., reversed-phase (RP) SPME)^13,47^ or ionic interaction (i.e., ion exchange SPME).^45,46^ After that, peptides can be eluted from SPME, followed by CZE-MS. Therefore, SPME has the potential to solve the low sample loading capacity issue of CZE completely.

The first applications of SPME-CZE-MS for peptides were performed in the 1990s.^48−50^ The system employed RP-SPME to improve the sample loading capacity of CZE-MS for highly sensitive peptide measurement. RP-SPME-CZE-MS can also be operated in a specific way to carry out two-dimensional separations via stepwise elution of peptides from SPME.^13,50^ Many reports on SPME-CZE-MS for bottom-up proteomics have been published in the past decade.^51^ However, there is no report on evaluating online SMPE-CZE-MS/MS toward the application of proteomic analysis of single human cells.

In this work, we built a simple and efficient RP-SPME part within the CZE separation capillary and coupled the RP-SMPE-CZE to a Q-Exactive HF mass spectrometer via an electrokinetically pumped sheath flow CE-MS interface.^52,53^ For the first time, we evaluated SPME-CZE-MS/MS for bottom-up proteomics of trace human cell proteome digests toward SCP applications. The system identified about 260 proteins using a Q-Exactive HF mass spectrometer when only 0.25 ng of a commercial HeLa cell lysate digest was available in the sample vial, and 0.1 ng of the sample was injected.

## Experimental Section

### Materials and Reagents

Hydrofluoric acid (HF, 48–51% solution in H_2_O) and acrylamide were purchased from Acros Organics (NJ, USA). Ammonium acetate (NH_4_CH_3_CO_2_) was purchased from Invitrogen (Thermo Fisher Scientific). LC/MS grade water, acetonitrile (ACN), methanol, and formic acid (FA) were purchased from Fisher Scientific (Pittsburgh, PA). LC/MS grade ammonium hydroxide (Ontario, CA) and 2-propanol (isopropanol) were purchased from Fisher Chemical (NJ, USA). Hydrochloric acid was purchased from Thermo Fisher Scientific. Sodium hydroxide (pellets) was purchased from Macron Fine Chemicals. Fused silica capillaries (50 μm i.d./360 μm o.d.) were obtained from Polymicro Technologies (Phoenix, AZ). The HeLa protein digest standard was purchased from Thermo Scientific. Aqueous mixtures were filtered with Nalgene Rapid-Flow Filter units (Thermo Scientific) with a 0.2 μm CN membrane and 50 mm diameter. Fused silica capillaries (50 μm i.d./360 μm o.d.) were obtained from Polymicro Technologies (Phoenix, AZ).

### Capillary Pretreatment and SPME Preparation

The linear polyacrylamide (LPA) coating was performed in fused silica capillaries (∼80 cm long) as previously described.^54^ The SPME was integrated into the capillary by packing C8 beads (1.9 μm, 120 Å, Dr. Maisch) into the CZE capillary with a premade frit (Frit Kit, Next Advance) in the sample injection end of the capillary. Briefly, the frit solution was prepared; one end of the capillary was submerged briefly in the solution, and the frit solution flowed inside (∼3 mm in length). A syringe filled with air was used to push the frit solution a few millimeters inside the capillary to make space for the beads. The frit was polymerized following the kit instructions. After this, a slurry of methanol and C8 beads was prepared and packed into the capillary with a syringe. The final length of the bead column was ∼2 mm.

### HeLa Protein Digest Standard Preparation

To test the performance of our SPME system, the following commercialized HeLa protein digest dilutions were prepared: 0.05, 0.25, 0.5, and 5 ng/μL. 5% acetic acid (AA) was used to reconstitute the peptides. Two μL of each solution was injected into the SPME system, resulting in 0.1, 0.5, 1.0 and 10 ng injections, respectively.

### SPME Operation

**Figure 1:**
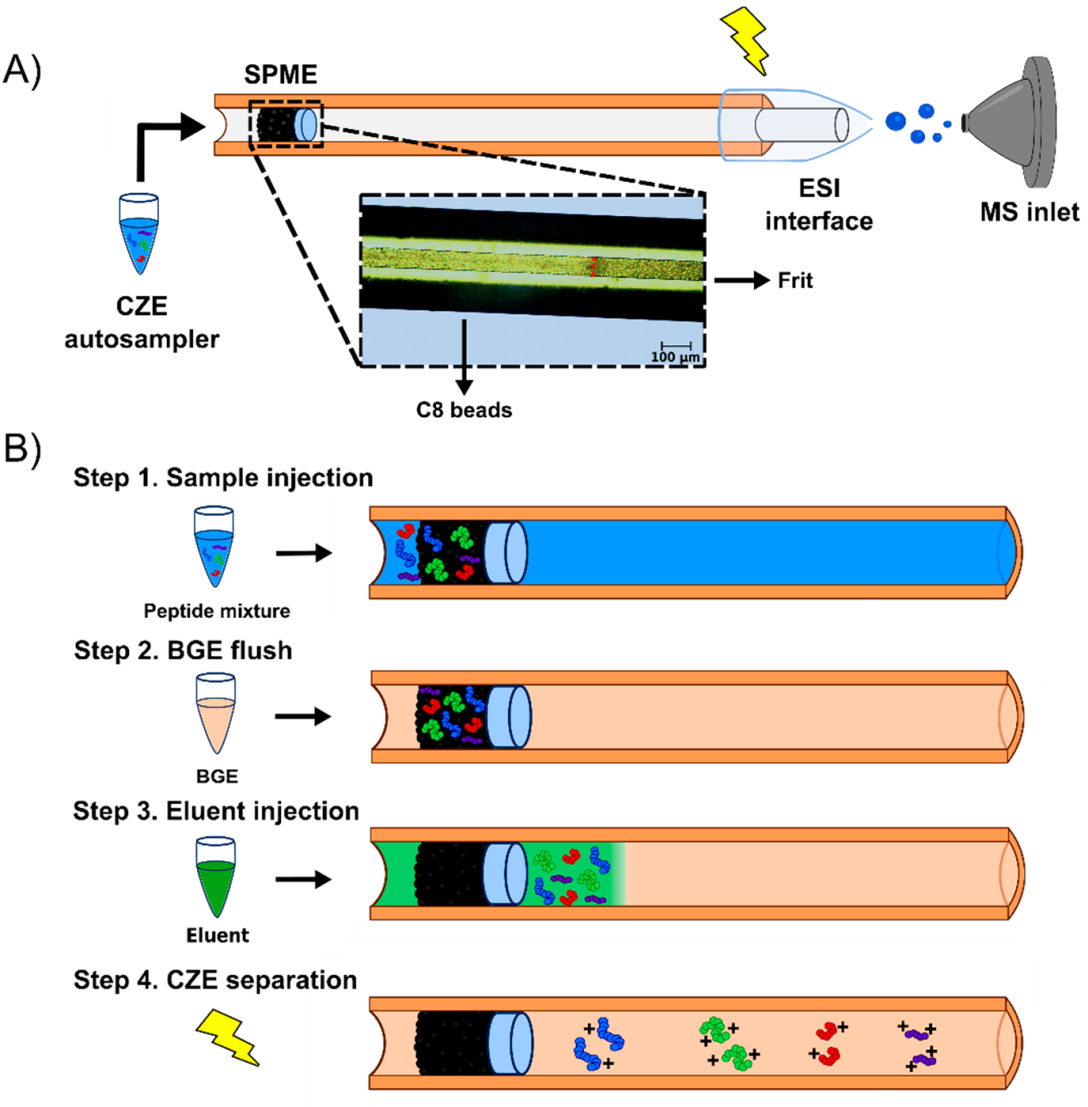
Schematic of the SPME-CZE platform and sample injection steps. (A) Schematic of the SPME-CZE system. The SPME is in the sample injection end of the capillary and consists of a premade frit and C8 column (∼2 mm long). The blue rectangle shows the SPME under the microscope at 10× (red dashed line shows the interface between the frit and beads). The electrospray ionization (ESI) interface used was an electrokinetically pumped sheath flow interface. (B) The first step for SPME analysis was sample injection into the system, followed by BGE flushing. The retained peptides were then eluted from the beads by using a small plug of elution buffer. Voltage was then applied to begin the CZE separation.

Samples were pressure-injected into the SPME as shown in Figure 1. Briefly, 5 μL of sample was dispensed in the CZE autosampler sample vial with a micropipette. Then, 2 μL of the sample was injected into the SPME by applying 60 psi for ∼15 min. Afterward, the capillary was flushed with background electrolyte (BGE) for approximately 20 min to remove any unretained peptide from the beads. Finally, a small plug (∼100 nL) of elution buffer (30% ACN, 50 mM ammonium acetate, pH 6.5) was injected to elute the peptides from the beads. Thirty kV was then applied for CZE separation.

### CZE and MS Parameters

A Beckman Coulter CESI 8000 Plus CE system (Sciex) was used for the CZE separation. A 5% AA solution was used as the background electrolyte (BGE). A solution of 10% methanol and 0.2% formic acid was used as sheath buffer. Thirty kV was applied at the injection end for CZE separation, and 2.2 kV was applied on the sheath buffer for ESI. An electrospray emitter with a 20–30 μm opening was pulled with a Sutter P-1000 flaming/brown micropipette puller. The third-generation electrokinetically pumped sheath flow CE-MS interface (EMASSII CE-MS interface, CMP Scientific)^52,53^ was used to couple CZE to MS.

A Q-Exactive HF (Thermo Fisher Scientific) mass spectrometer was used in all of the experiments. For full MS acquisition, the resolution was set to 60,000 AGC target to 3e6, maximum injection time (IT) to 50 ms, and a scan range of 300–1500 *m*/*z*. For MS/MS, the AGC target was set to 1e5 and the maximum IT to 100 ms. A Top10 data-dependent acquisition (DDA) method was applied. Dynamic exclusion was set to 15 s, and MS/MS intensity threshold was set to 2.0e4.

### Database Search

Proteome Discoverer 2.2 software (Thermo Fisher Scientific) with a Sequest HT search engine was used for database search. HeLa samples were searched against the *Homo sapiens* UniProt database proteome (UP000005640). Database search parameters were changed as follows: precursor and product ion mass tolerances were set to 20 ppm and 0.05 Da, respectively. The digestion enzyme was set to trypsin. Oxidation of methionine, deamidation of asparagine and glutamine, and acetylation of the protein N-terminal were set as dynamic modifications. Carbamidomethylation of cysteine was set as a fixed modification. The false discovery rate (FDR) was performed with the target-decoy database approach. Peptides and peptide spectrum matches (PSMs) were filtered with a 1% FDR. The identified proteins are listed in Supporting Information.

MaxQuant 1.5.5.1 was also used for a database search to perform label-free quantification (LFQ). The match between runs (MBR) algorithm was also enabled with parameters set as the default. *Homo sapiens* UniProt database proteome (UP000005640) was used for database search. Trypsin and LysC were set as the digestion enzymes. The dynamic modifications set in Proteome Discoverer 2.2 were set as variable modifications in MaxQuant. Carbamidomethylation of cysteine was set as a fixed modification. Common contaminants were included. A 1% FDR was used to filter the identified PSMs, peptides, and protein groups.

## Results and Discussion

The goal is to develop an RP-SPME-CZE-MS/MS system to boost the performance of CZE-MS/MS for the bottom-up proteomics analysis of extremely mass-limited samples (i.e., picograms of HeLa cell lysate digests). For construction of SPME-CZE, we made a frit close to the sample injection end of the CZE separation capillary via in situ polymerization and packed C8 beads into the capillary, creating an about 2 mm long SPME part. If needed, the beads could be removed and repacked easily. Some preliminary work in our SPME system development showed that using C8 beads instead of the commonly used C18 beads provided much better peptide recovery from the beads (data not shown). Therefore, we employed C8 beads in this study. For this project, we put extremely low masses of the commercial HeLa cell lysate digest into sample vials, corresponding to 0.25–25 ng of peptides. The sample vials were treated by bovine serum albumin to reduce sample loss due to adsorption according to our previous study.^55^ The system was able to load 40% of the available peptide material in each sample vial for each measurement, corresponding to the injection of 0.1–10 ng of the HeLa cell digest. If we assume each HeLa cell contains roughly 250 pg proteins,^56,57^ the mass of available peptide material in each sample vial corresponds to roughly 1–100 cells. The mass of injected peptides equals that in only 0.4–40 cells.

**Figure 2:**
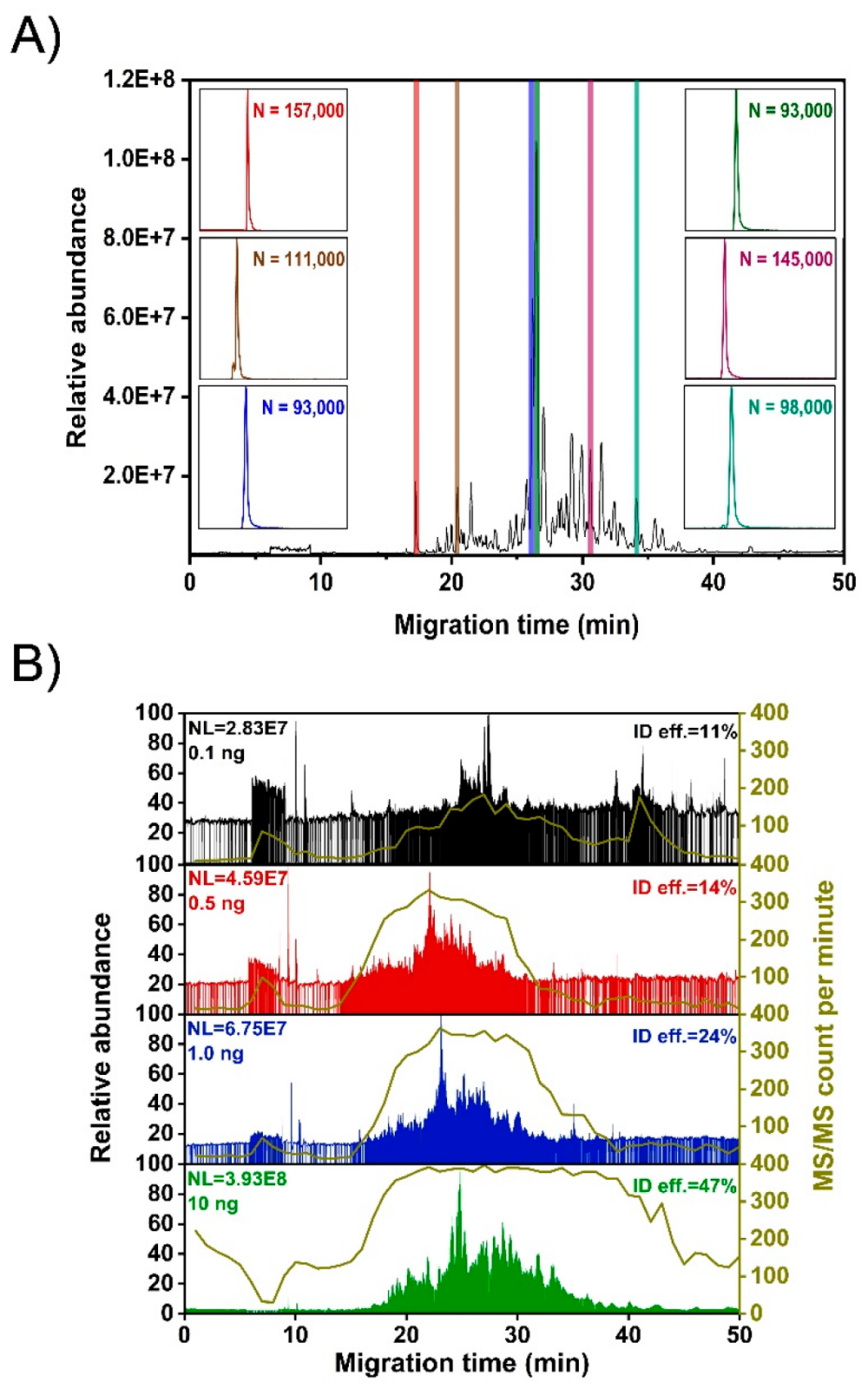
(A) Base peak electropherogram of the HeLa cell digest (10 ng injection). Extracted ion electropherograms (EIEs) of six peptides with a calculated number of theoretical plates (*N*) are shown. (B) Total ion current electropherograms of picograms to low-ng HeLa digest samples. MS/MS information was obtained from MaxQuant with the MBR function. The identification efficiency for each run is shown. MS/MS count per minute distribution is shown with a brown line.

The SPME-CZE system produced a high efficiency for peptide separations. As shown in Figure 2A, the example peptides across the CE-MS run have over 90,000 theoretical plates. The platform acquired a reasonably high number of MS/MS spectra (3,500) when only 0.1 ng of HeLa cell digest was loaded into the system from a sample vial containing only 0.25 ng of peptides. When the sample loading amount increased from 0.1 to 10 ng, the number of acquired MS/MS spectra was boosted by 4.5-fold due to the substantially higher peptide intensity for triggering MS/MS acquisition. The identification efficiency, defined as the ratio between the number of PSMs and MS/MS spectra, increased from 11% with the 0.1 ng injection to 47% with 10 ng injection, Figure 2B. Most of the peptides migrated out of the CZE capillary between 10 and 50 min. According to the number of MS/MS per minute distribution data, peptides were concentrated within 20–35 min. The data show that our SPME-CZE-MS system has the capacity to efficiently capture, elute, separate, and measure a complex mixture of peptides even when the total mass is in the range of low ng to pg.

**Figure 3:**
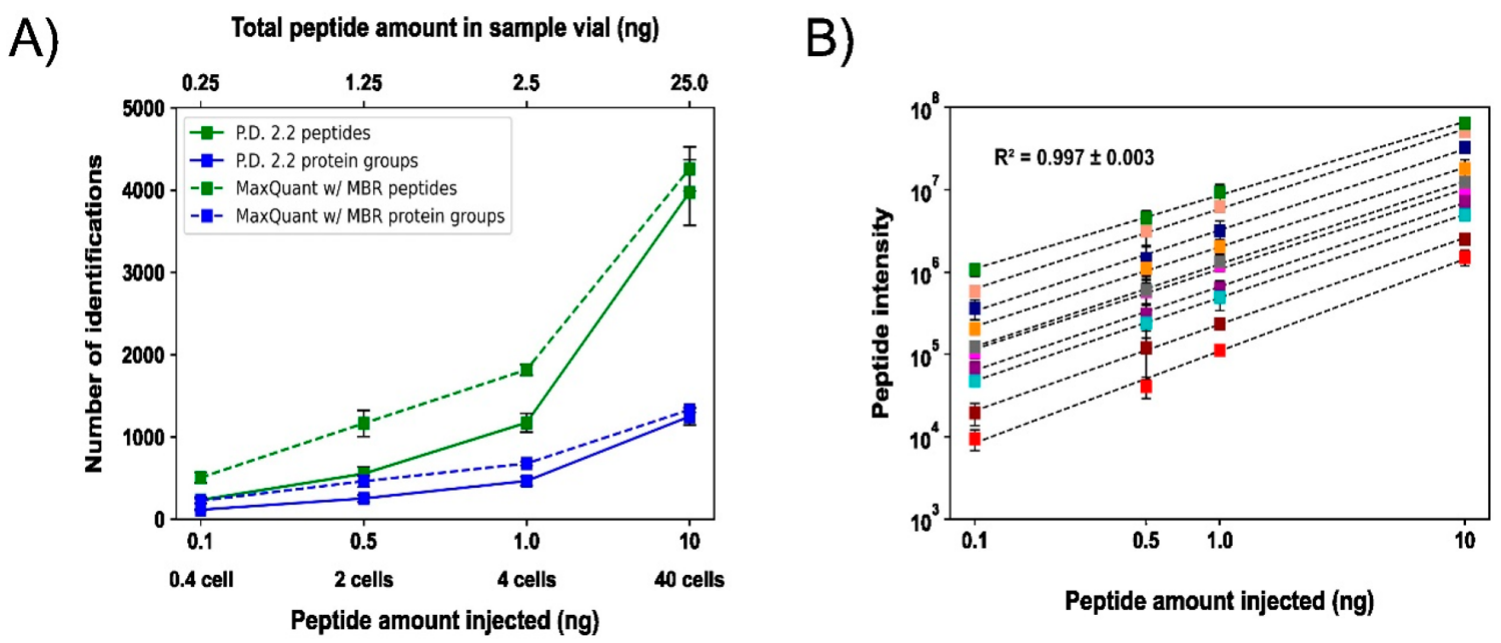
Summary of the peptide and protein identifications. (A) Peptide and protein group identifications from 0.25 to 25 ng of the commercialized HeLa digest samples. The injected sample amount ranges from 0.1 to 10 ng, corresponding to the protein mass of 0.4 to 40 HeLa cells. Dashed lines represent the data from MaxQuant with MBR. Solid lines represent identifications obtained from Proteome Discoverer 2.2. The axis at the bottom shows the sample amount injected and the equivalent in terms of the number of HeLa cells. On top, the total amount of peptides in the sample vial is labeled. The error bars represent the standard deviation (SD) of the number of peptide and protein identifications from duplicate measurements. (B) Log–log plots of peptide intensity as a function of injected peptide amount in a range of 0.1–10 ng. Ten randomly selected peptides were used for the plots. The mean and SD of Pearson correlation coefficients (*R*^2^) of the ten peptides are labeled. The error bars represent the SD of the peptide intensity from the duplicate measurements.

As shown in Figure 3A, the number of peptide and protein group identifications increased significantly as the peptide injection amount was boosted from 0.1 to 10 ng, corresponding to the protein mass of 0.4 to 40 HeLa cells. For the 0.1 ng injection, equivalent to the amount of protein in less than half a HeLa cell, 117 ± 8 protein groups and 236 ± 40 peptides (*n* = 2) were identified by the SPME-CZE-MS/MS with the Proteome Discoverer 2.2 software. The number of peptide and protein group identifications doubled when the MaxQuant software and MBR algorithm were used (523 ± 69 peptides and 257 ± 24 protein groups). It has been reported that over 700 proteins could be identified by CZE-MS/MS using an Orbitrap Fusion Lumos Tribrid mass spectrometer from HeLa cell digests when only a single-cell level mass of peptides was injected.^32^ However, the measurement required a peptide material corresponding to hundreds of HeLa cells available in the sample vial, further demonstrating the challenge for doing real SCP of human cells using CZE-MS/MS. In our study, we demonstrate the feasibility of SCP using SPME-CZE-MS/MS for the first time, identifying hundreds of proteins using a Q-Exactive HF mass spectrometer when only 0.25 ng of a HeLa cell digest (about the protein mass of one HeLa cell) is available for measurement. We expect that coupling the SPME-CZE to a high-end mass spectrometer with substantially better sensitivity and scan speed than that of Q-Exactive HF will boost the number of protein and peptide identifications dramatically.

When 0.5 ng of the HeLa cell digest was loaded from a 1.25-ng sample in the vial, 512 proteins and 1199 peptides were identified using MaxQuant and MBR, Figure 3A. The data show about a 5-fold increase in the number of protein identifications compared to our previous dynamic pH junction-based CZE-MS/MS data^58^ when only roughly 1 ng of a human cell proteome digest is available in the sample vial for measurement. Both studies employed similar CZE and MS conditions. The data further document the value of SPME-CZE-MS/MS for mass-limited samples compared to typical CZE-MS/MS. We further studied the correlation between peptide intensity and the injected peptide amount from 0.1 to 10 ng, Figure 3B. We observed linear correlations for ten randomly selected peptides with the *R*^2^ better than 0.992. The data suggests that the SPME-CZE-MS/MS system is quantitative.

CZE-MS not only offers highly efficient peptide separation and highly sensitive peptide detection but also provides an opportunity for validating peptide identifications from the target-decoy database search using the correlation between experimental and theoretical electrophoretic mobility (μ_ef_) of identified peptides. The μ_ef_ of identified peptides from CZE-MS/MS can be predicted accurately according to literature reports.^59,60^ We can provide another level of data validation by correlating the experimental and theoretical μ_ef_ values of peptides. This is particularly useful for the data of single cells due to their relatively lower peptide and fragment ion intensities compared to regular bulk measurements. Here, to further validate the confidence of peptide identifications and avoid false positives in the low-ng and pg HeLa cell digest samples, we calculated the experimental and theoretical μ_ef_ (m^2^ V^–1^ s^–1^) of the peptides according to eqs 1 and 2, respectively
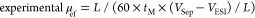
1where *L* is the capillary length in meters, *t*_M_ is the retention time in minutes, *V*_Sep_ is the voltage applied for separation in V, and *V*_ESI_ is the voltage applied for electrospray ionization in V.

To calculate the theoretical μ_ef_, we used a semiempirical predicting model reported in the literature^61^
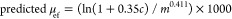
2where *c* and *m* are the peptide’s charge and mass, respectively.

**Figure 4:**
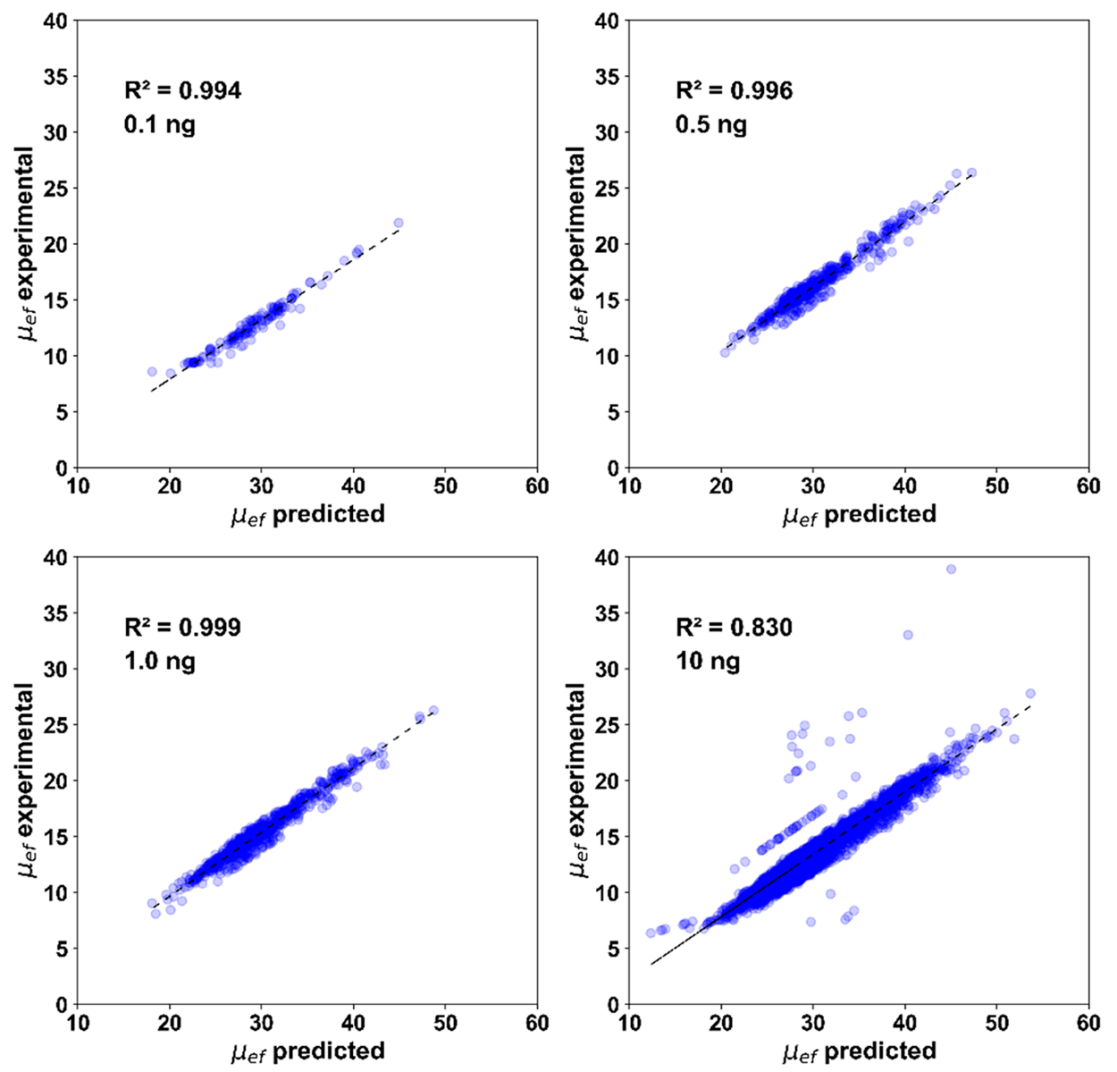
Correlations between experimental and theoretical μ_ef_ values of identified peptides from 0.1, 0.5, 1.0, and 10 ng injections. Each graph represents one of the replicates taken from each sample. The unit of μ_ef_ is m^2^ V^–1^ s^–1^. The Pearson correlation coefficients (*R*^2^) are labeled.

We observed linear correlations between predicted and experimental μ_ef_ for 0.1–10 ng injection data (*R*^2^ > 0.99 for 0.1, 0.5 and 1.0 ng data; *R*^2^ = 0.83 for the 10 ng data), Figure 4. Some peptides are off the main trend due to potential false positive identifications. The data demonstrate the high confidence of the peptide identifications from 0.1 to 10 ng of HeLa cell proteome digests. The accurate prediction of peptides’ μ_ef_ renders CZE-MS/MS an excellent technique for bottom-up proteomics because the μ_ef_ information could be useful for boosting the performance of database search for more peptide and protein identifications with high confidence.

## Conclusions

We successfully developed an RP-SPME-CZE-MS/MS system that can efficiently capture and analyze low-ng and -pg levels of peptide material from a commercialized HeLa digest. Hundreds of proteins were identified by the SPME-CZE-MS/MS system (Q-Exactive HF) when only a picogram amount of HeLa digest is available in the sample vial for measurement. The results indicate the capability of the technique as an alternative to RPLC-MS/MS for the bottom-up proteomics of single human cells. We expect that the number of peptide and protein identifications from the trace amount of human cell digest can be boosted substantially via coupling the RP-SPME-CZE system to a high-end mass spectrometer (i.e., timsTOF, Orbitrap Fusion Lumos, and Orbitrap Astral) with much higher sensitivity and scan rate compared with the Q-Exactive HF used in this study.

SPME-CZE-MS typically has issues related to reproducibility and robustness due to bubble formation in the SPME part under an electric field and pressure. In this pilot study, we made significant efforts to optimize the operations and composition of the elution buffer to minimize the bubble formation issue, producing reasonable reproducibility. However, we did not study the capillary-to-capillary reproducibility. Since we eluted peptides from the SPME using a relatively large volume of elution buffer (100 nL, 30% ACN, 50 mM ammonium acetate, pH 6.5), we did not observe significant peptide carry-over. More investigations are needed to make the technique ready for broad applications, i.e., long-term reproducibility, capillary-to-capillary reproducibility, and capability for measuring real single-cell samples.

We need to point out that our SPME-CZE-MS/MS technique needs to be coupled with advanced sample preparation techniques^5,62−64^ for proteomics characterization of single human cells. Those cutting-edge sample preparation techniques typically carry out sample preparation of single cells in a sub-μL to low μL volume. The single-cell sample with a low μL volume can be directly analyzed by SPME-CZE-MS/MS, and the sample with a sub-μL volume can be mixed with an MS-compatible buffer (i.e., 5% acetic acid) to reach a volume of 1–2 μL before SPME-CZE-MS/MS analysis. The MS raw data has been deposited to the ProteomeXchange Consortium via the PRIDE partner repository^65^ with the data set identifier PXD047627.

## Supporting Information Available

Lists of protein groups identified by SPME-CZE-MS/MS from 0.1 to 10 ng of HeLa cell digests (XLSX)

## References

[ref1] Sakamoto W.; Azegami N.; Konuma T.; Akashi S. Single-Cell Native Mass Spectrometry of Human Erythrocytes. Anal. Chem. 2021, 93 (17), 6583–6588. 10.1021/acs.analchem.1c00588. 33871982

[ref2] Johnson K. R.; Greguš M.; Ivanov A. R. Coupling High-Field Asymmetric Ion Mobility Spectrometry with Capillary Electrophoresis-Electrospray Ionization-Tandem Mass Spectrometry Improves Protein Identifications in Bottom-Up Proteomic Analysis of Low Nanogram Samples. J. Proteome Res. 2022, 21 (10), 2453–2461. 10.1021/acs.jproteome.2c00337. 36112031 PMC10118849

[ref3] Budnik B.; Levy E.; Harmange G.; Slavov N. SCoPE-MS: mass spectrometry of single mammalian cells quantifies proteome heterogeneity during cell differentiation. Genome Biol. 2018, 19 (1), 161 10.1186/s13059-018-1547-5. 30343672 PMC6196420

[ref4] Johnston S. M.; Webber K. G. I.; Xie X.; Truong T.; Nydegger A.; Lin H.-J. L.; Nwosu A.; Zhu Y.; Kelly R. T. Rapid, One-Step Sample Processing for Label-Free Single-Cell Proteomics. J. Am. Soc. Mass Spectrom. 2023, 34 (8), 1701–1707. 10.1021/jasms.3c00159. 37410391 PMC11017373

[ref5] Zhu Y.; Piehowski P. D.; Zhao R.; Chen J.; Shen Y.; Moore R. J.; Shukla A. K.; Petyuk V. A.; Campbell-Thompson M.; Mathews C. E.; Smith R. D.; Qian W. J.; Kelly R. T. Nanodroplet processing platform for deep and quantitative proteome profiling of 10–100 mammalian cells. Nat. Commun. 2018, 9 (1), 882 10.1038/s41467-018-03367-w. 29491378 PMC5830451

[ref6] Zhao P.; Feng Y.; Wu J.; Zhu J.; Yang J.; Ma X.; Ouyang Z.; Zhang X.; Zhang W.; Wang W. Efficient Sample Preparation System for Multi-Omics Analysis via Single Cell Mass Spectrometry. Anal. Chem. 2023, 95 (18), 7212–7219. 10.1021/acs.analchem.2c05728. 37078759

[ref7] Wojtkiewicz M.; Berg Luecke L.; Castro C.; Burkovetskaya M.; Mesidor R.; Gundry R. L. Bottom-up proteomic analysis of human adult cardiac tissue and isolated cardiomyocytes. J. Mol. Cell. Cardiol. 2022, 162, 20–31. 10.1016/j.yjmcc.2021.08.008. 34437879 PMC9620472

[ref8] Yao H.; Zhao H.; Zhao X.; Pan X.; Feng J.; Xu F.; Zhang S.; Zhang X. Label-free Mass Cytometry for Unveiling Cellular Metabolic Heterogeneity. Anal. Chem. 2019, 91 (15), 9777–9783. 10.1021/acs.analchem.9b01419. 31242386

[ref9] Park J.; Yu F.; Fulcher J. M.; Williams S. M.; Engbrecht K.; Moore R. J.; Clair G. C.; Petyuk V.; Nesvizhskii A. I.; Zhu Y. Evaluating Linear Ion Trap for MS3-Based Multiplexed Single-Cell Proteomics. Anal. Chem. 2023, 95 (3), 1888–1898. 10.1021/acs.analchem.2c03739. PMC12964444 36637389

[ref10] Johnson K. R.; Gao Y.; Greguš M.; Ivanov A. R. On-capillary Cell Lysis Enables Top-down Proteomic Analysis of Single Mammalian Cells by CE-MS/MS. Anal. Chem. 2022, 94 (41), 14358–14367. 10.1021/acs.analchem.2c03045. 36194750 PMC10118848

[ref11] Truong T.; Webber K. G. I.; Madisyn Johnston S.; Boekweg H.; Lindgren C. M.; Liang Y.; Nydegger A.; Xie X.; Tsang T.; Jayatunge D. A. D. N.; Andersen J. L.; Payne S. H.; Kelly R. T. Data-Dependent Acquisition with Precursor Coisolation Improves Proteome Coverage and Measurement Throughput for Label-Free Single-Cell Proteomics. Angew. Chem., Int. Ed. 2023, 62 (34), e202303415 10.1002/anie.202303415. PMC10529037 37380610

[ref12] Lombard-Banek C.; Moody S. A.; Manzini M. C.; Nemes P. Microsampling Capillary Electrophoresis Mass Spectrometry Enables Single-Cell Proteomics in Complex Tissues: Developing Cell Clones in Live Xenopus laevis and Zebrafish Embryos. Anal. Chem. 2019, 91 (7), 4797–4805. 10.1021/acs.analchem.9b00345. 30827088 PMC6688183

[ref13] Wang Y.; Fonslow B. R.; Wong C. C. L.; Nakorchevsky A.; Yates J. R 3rd. Improving the comprehensiveness and sensitivity of sheathless capillary electrophoresis-tandem mass spectrometry for proteomic analysis. Anal. Chem. 2012, 84 (20), 8505–8513. 10.1021/ac301091m. 23004022 PMC3498465

[ref14] Zhang Z.; Dubiak K. M.; Huber P. W.; Dovichi N. J. Miniaturized Filter-Aided Sample Preparation (MICRO-FASP) Method for High Throughput, Ultrasensitive Proteomics Sample Preparation Reveals Proteome Asymmetry in Xenopus laevis Embryos. Anal. Chem. 2020, 92 (7), 5554–5560. 10.1021/acs.analchem.0c00470. 32125139 PMC7931810

[ref15] Hughes C. S.; Moggridge S.; Müller T.; Sorensen P. H.; Morin G. B.; Krijgsveld J. Single-pot, solid-phase-enhanced sample preparation for proteomics experiments. Nat. Protoc. 2019, 14 (1), 68–85. 10.1038/s41596-018-0082-x. 30464214

[ref16] Yang Z.; Sun L. Recent technical progress in sample preparation and liquid-phase separation-mass spectrometry for proteomic analysis of mass-limited samples. Anal. Methods. 2021, 13 (10), 1214–1225. 10.1039/D1AY00171J. 33629703

[ref17] Vistain L. F.; Tay S. Single-Cell Proteomics. Trends Biochem. Sci. 2021, 46 (8), 661–672. 10.1016/j.tibs.2021.01.013. 33653632 PMC11697639

[ref18] Labib M.; Kelley S. O. Single-cell analysis targeting the proteome. Nat. Rev. Chem. 2020, 4 (3), 143–158. 10.1038/s41570-020-0162-7. 37128021

[ref19] Toby T. K.; Fornelli L.; Kelleher N. L. Progress in Top-Down Proteomics and the Analysis of Proteoforms. Annu. Rev. Anal. Chem. 2016, 9 (1), 499–519. 10.1146/annurev-anchem-071015-041550. PMC5373801 27306313

[ref20] Smith L. M.; Kelleher N. L. Proteoform: a single term describing protein complexity. Nat. Methods. 2013, 10 (3), 186–187. 10.1038/nmeth.2369. 23443629 PMC4114032

[ref21] Chen D.; McCool E. N.; Yang Z.; Shen X.; Lubeckyj R. A.; Xu T.; Wang Q.; Sun L. Recent advances (2019–2021) of capillary electrophoresis-mass spectrometry for multilevel proteomics. Mass Spectrom. Rev. 2023, 42 (2), 617–642. 10.1002/mas.21714. 34128246 PMC8671558

[ref22] Duong V. A.; Park J. M.; Lee H. Review of Three-Dimensional Liquid Chromatography Platforms for Bottom-Up Proteomics. Int. J. Mol. Sci. 2020, 21 (4), 1524 10.3390/ijms21041524. 32102244 PMC7073195

[ref23] Cong Y.; Liang Y.; Motamedchaboki K.; Huguet R.; Truong T.; Zhao R.; Shen Y.; Lopez-Ferrer D.; Zhu Y.; Kelly R. T. Improved Single-Cell Proteome Coverage Using Narrow-Bore Packed NanoLC Columns and Ultrasensitive Mass Spectrometry. Anal. Chem. 2020, 92 (3), 2665–2671. 10.1021/acs.analchem.9b04631. 31913019 PMC7550239

[ref24] Lombard-Banek C.; Moody S. A.; Nemes P. Single-Cell Mass Spectrometry for Discovery Proteomics: Quantifying Translational Cell Heterogeneity in the 16-Cell Frog (Xenopus) Embryo. Angew. Chem., Int. Ed. 2016, 55 (7), 2454–2458. 10.1002/anie.201510411. PMC4755155 26756663

[ref25] Choi S. B.; Lombard-Banek C.; Muñoz-LLancao P.; Manzini M. C.; Nemes P. Enhanced Peptide Detection Toward Single-Neuron Proteomics by Reversed-Phase Fractionation Capillary Electrophoresis Mass Spectrometry. J. Am. Soc. Mass Spectrom. 2018, 29 (5), 913–922. 10.1007/s13361-017-1838-1. 29147852

[ref26] Choi S. B.; Zamarbide M.; Manzini M. C.; Nemes P. Tapered-Tip Capillary Electrophoresis Nano-Electrospray Ionization Mass Spectrometry for Ultrasensitive Proteomics: the Mouse Cortex. J. Am. Soc. Mass Spectrom. 2017, 28 (4), 597–607. 10.1007/s13361-016-1532-8. 27853976

[ref27] Ludwig K. R.; Sun L.; Zhu G.; Dovichi N. J.; Hummon A. B. Over 2300 phosphorylated peptide identifications with single-shot capillary zone electrophoresis-tandem mass spectrometry in a 100 min separation. Anal. Chem. 2015, 87 (19), 9532–9537. 10.1021/acs.analchem.5b02457. 26399161 PMC4605816

[ref28] Zhu G.; Sun L.; Yan X.; Dovichi N. J. Single-shot proteomics using capillary zone electrophoresis-electrospray ionization-tandem mass spectrometry with production of more than 1250 Escherichia coli peptide identifications in a 50 min separation. Anal. Chem. 2013, 85 (5), 2569–2573. 10.1021/ac303750g. 23394296 PMC3594080

[ref29] Faserl K.; Sarg B.; Kremser L.; Lindner H. Optimization and evaluation of a sheathless capillary electrophoresis-electrospray ionization mass spectrometry platform for peptide analysis: comparison to liquid chromatography-electrospray ionization mass spectrometry. Anal. Chem. 2011, 83 (19), 7297–7305. 10.1021/ac2010372. 21848273

[ref30] Amenson-Lamar E. A.; Sun L.; Zhang Z.; Bohn P. W.; Dovichi N. J. Detection of 1 zmol injection of angiotensin using capillary zone electrophoresis coupled to a Q-Exactive HF mass spectrometer with an electrokinetically pumped sheath-flow electrospray interface. Talanta. 2019, 204, 70–73. 10.1016/j.talanta.2019.05.079. 31357355 PMC6668918

[ref31] Choi S. B.; Polter A. M.; Nemes P. Patch-Clamp Proteomics of Single Neurons in Tissue Using Electrophysiology and Subcellular Capillary Electrophoresis Mass Spectrometry. Anal. Chem. 2022, 94 (3), 1637–1644. 10.1021/acs.analchem.1c03826. 34964611

[ref32] Johnson K. R.; Greguš M.; Kostas J. C.; Ivanov A. R. Capillary Electrophoresis Coupled to Electrospray Ionization Tandem Mass Spectrometry for Ultra-Sensitive Proteomic Analysis of Limited Samples. Anal. Chem. 2022, 94 (2), 704–713. 10.1021/acs.analchem.1c02929. 34983182 PMC8770592

[ref33] Sun L.; Zhu G.; Zhao Y.; Yan X.; Mou S.; Dovichi N. J. Ultrasensitive and fast bottom-up analysis of femtogram amounts of complex proteome digests. Angew. Chem., Int. Ed. 2013, 52 (51), 13661–13664. 10.1002/anie.201308139. PMC3904452 24173663

[ref34] Zhu G.; Sun L.; Yan X.; Dovichi N. J. Bottom-up proteomics of Escherichia coli using dynamic pH junction preconcentration and capillary zone electrophoresis-electrospray ionization-tandem mass spectrometry. Anal. Chem. 2014, 86 (13), 6331–6336. 10.1021/ac5004486. 24852005 PMC4082393

[ref35] Chen D.; Shen X.; Sun L. Capillary zone electrophoresis-mass spectrometry with microliter-scale loading capacity, 140 min separation window and high peak capacity for bottom-up proteomics. Analyst. 2017, 142 (12), 2118–2127. 10.1039/C7AN00509A. 28513658

[ref36] Britz-McKibbin P.; Chen D. D. Y. Selective focusing of catecholamines and weakly acidic compounds by capillary electrophoresis using a dynamic pH junction. Anal. Chem. 2000, 72 (6), 1242–1252. 10.1021/ac990898e. 10740866

[ref37] Wang L.; Cheng J.; McNutt J. E.; Morin G. B.; Chen D. D. Y. Dynamic pH barrage junction focusing of amino acids, peptides, and digested monoclonal antibodies in capillary electrophoresis-mass spectrometry. Electrophoresis. 2020, 41 (21–22), 1832–1842. 10.1002/elps.202000076. 32436592

[ref38] Zhang Z.; Hebert A. S.; Westphall M. S.; Coon J. J.; Dovichi N. J. Single-Shot Capillary Zone Electrophoresis-Tandem Mass Spectrometry Produces over 4400 Phosphopeptide Identifications from a 220 ng Sample. J. Proteome Res. 2019, 18 (8), 3166–3173. 10.1021/acs.jproteome.9b00244. 31180221 PMC6679793

[ref39] Guo X.; Fillmore T. L.; Gao Y.; Tang K. Capillary Electrophoresis-Nanoelectrospray Ionization-Selected Reaction Monitoring Mass Spectrometry via a True Sheathless Metal-Coated Emitter Interface for Robust and High-Sensitivity Sample Quantification. Anal. Chem. 2016, 88 (8), 4418–4425. 10.1021/acs.analchem.5b04912. 27028594 PMC4854437

[ref40] Shen Y.; Berger S. J.; Anderson G. A.; Smith R. D. High-efficiency capillary isoelectric focusing of peptides. Anal. Chem. 2000, 72 (9), 2154–2159. 10.1021/ac991367t. 10815979

[ref41] Paša-Tolić L.; Jensen P. K.; Anderson G. A.; Lipton M. S.; Peden K. K.; Martinović S.; Tolić N.; Bruce J. E.; Smith R. D. High Throughput Proteome-Wide Precision Measurements of Protein Expression Using Mass Spectrometry. J. Am. Chem. Soc. 1999, 121 (34), 7949–7950. 10.1021/ja991063o.

[ref42] Kachman M. T.; Wang H.; Schwartz D. R.; Cho K. R.; Lubman D. M. A 2-D liquid separations/mass mapping method for interlysate comparison of ovarian cancers. Anal. Chem. 2002, 74 (8), 1779–1791. 10.1021/ac011159c. 11985308

[ref43] Xu T.; Shen X.; Yang Z.; Chen D.; Lubeckyj R. A.; McCool E. N.; Sun L. Automated Capillary Isoelectric Focusing-Tandem Mass Spectrometry for Qualitative and Quantitative Top-Down Proteomics. Anal. Chem. 2020, 92 (24), 15890–15898. 10.1021/acs.analchem.0c03266. 33263984 PMC8564864

[ref44] Shen X.; Liang Z.; Xu T.; Yang Z.; Wang Q.; Chen D.; Pham L.; Du W.; Sun L. Investigating native capillary zone electrophoresis-mass spectrometry on a high-end quadrupole-time-of-flight mass spectrometer for the characterization of monoclonal antibodies. Int. J. Mass Spectrom. 2021, 462, 116541 10.1016/j.ijms.2021.116541. 33642939 PMC7906288

[ref45] Zhang Z.; Yan X.; Sun L.; Zhu G.; Dovichi N. J. Detachable strong cation exchange monolith, integrated with capillary zone electrophoresis and coupled with pH gradient elution, produces improved sensitivity and numbers of peptide identifications during bottom-up analysis of complex proteomes. Anal. Chem. 2015, 87 (8), 4572–4577. 10.1021/acs.analchem.5b00789. 25822566 PMC4406858

[ref46] Zhang Z.; Sun L.; Zhu G.; Yan X.; Dovichi N. J. Integrated strong cation-exchange hybrid monolith coupled with capillary zone electrophoresis and simultaneous dynamic pH junction for large-volume proteomic analysis by mass spectrometry. Talanta. 2015, 138, 117–122. 10.1016/j.talanta.2015.01.040. 25863379 PMC4394190

[ref47] Medina-Casanellas S.; Domínguez-Vega E.; Benavente F.; Sanz-Nebot V.; Somsen G. W.; De Jong G. J. Low-picomolar analysis of peptides by on-line coupling of fritless solid-phase extraction to sheathless capillary electrophoresis-mass spectrometry. J. Chromatogr. A. 2014, 1328, 1–6. 10.1016/j.chroma.2013.12.080. 24438833

[ref48] Figeys D.; Ducret A.; Yates J. R. 3rd; Aebersold, R. Protein identification by solid phase microextraction-capillary zone electrophoresis-microelectrospray-tandem mass spectrometry. Nat. Biotechnol. 1996, 14 (11), 1579–1583. 10.1038/nbt1196-1579. 9634825

[ref49] Figeys D.; Corthals G. L.; Gallis B.; Goodlett D. R.; Ducret A.; Corson M. A.; Aebersold R. Data-dependent modulation of solid-phase extraction capillary electrophoresis for the analysis of complex peptide and phosphopeptide mixtures by tandem mass spectrometry: application to endothelial nitric oxide synthase. Anal. Chem. 1999, 71 (13), 2279–2287. 10.1021/ac9813991. 10405598

[ref50] Tong W.; Link A.; Eng J. K.; Yates J. R 3rd. Identification of proteins in complexes by solid-phase microextraction/multistep elution/capillary electrophoresis/tandem mass spectrometry. Anal. Chem. 1999, 71 (13), 2270–2278. 10.1021/ac9901182. 10405597

[ref51] Zhang Z.; Qu Y.; Dovichi N. J. Capillary zone electrophoresis-mass spectrometry for bottom-up proteomics. TrAC Trends Anal. Chem. 2018, 108, 23–37. 10.1016/j.trac.2018.08.008.

[ref52] Sun L.; Zhu G.; Zhang Z.; Mou S.; Dovichi N. J. Third-generation electrokinetically pumped sheath-flow nanospray interface with improved stability and sensitivity for automated capillary zone electrophoresis-mass spectrometry analysis of complex proteome digests. J. Proteome Res. 2015, 14 (5), 2312–2321. 10.1021/acs.jproteome.5b00100. 25786131 PMC4416984

[ref53] Wojcik R.; Dada O. O.; Sadilek M.; Dovichi N. J. Simplified capillary electrophoresis nanospray sheath-flow interface for high efficiency and sensitive peptide analysis. Rapid Commun. Mass Spectrom. 2010, 24 (17), 2554–2560. 10.1002/rcm.4672. 20740530

[ref54] McCool E. N.; Lubeckyj R.; Shen X.; Kou Q.; Liu X.; Sun L. Large-scale Top-down Proteomics Using Capillary Zone Electrophoresis Tandem Mass Spectrometry. J. Vis. Exp. 2018, 140, e58644 10.3791/58644-v. PMC6235596 30417888

[ref55] Yang Z.; Shen X.; Chen D.; Sun L. Improved Nanoflow RPLC-CZE-MS/MS System with High Peak Capacity and Sensitivity for Nanogram Bottom-up Proteomics. J. Proteome Res. 2019, 18 (11), 4046–4054. 10.1021/acs.jproteome.9b00545. 31610113 PMC7545725

[ref56] Cohen D.; Dickerson J. A.; Whitmore C. D.; Turner E. H.; Palcic M. M.; Hindsgaul O.; Dovichi N. J. Chemical cytometry: fluorescence-based single-cell analysis. Annu. Rev. Anal. Chem. 2008, 1 (1), 165–190. 10.1146/annurev.anchem.1.031207.113104. 20636078

[ref57] Dolgalev G. V.; Safonov T. A.; Arzumanian V. A.; Kiseleva O. I.; Poverennaya E. V. Estimating Total Quantitative Protein Content in Escherichia coli, Saccharomyces cerevisiae, and HeLa Cells. Int. J. Mol. Sci. 2023, 24 (3), 2081 10.3390/ijms24032081. 36768409 PMC9916689

[ref58] Yang Z.; Shen X.; Chen D.; Sun L. Microscale Reversed-Phase Liquid Chromatography/Capillary Zone Electrophoresis-Tandem Mass Spectrometry for Deep and Highly Sensitive Bottom-Up Proteomics: Identification of 7500 Proteins with Five Micrograms of an MCF7 Proteome Digest. Anal. Chem. 2018, 90 (17), 10479–10486. 10.1021/acs.analchem.8b02466. 30102516 PMC6156779

[ref59] Chen D.; Ludwig K. R.; Krokhin O. V.; Spicer V.; Yang Z.; Shen X.; Hummon A. B.; Sun L. Capillary Zone Electrophoresis-Tandem Mass Spectrometry for Large-Scale Phosphoproteomics with the Production of over 11,000 Phosphopeptides from the Colon Carcinoma HCT116 Cell Line. Anal. Chem. 2019, 91 (3), 2201–2208. 10.1021/acs.analchem.8b04770. 30624053 PMC6506858

[ref60] Krokhin O. V.; Anderson G.; Spicer V.; Sun L.; Dovichi N. J. Predicting Electrophoretic Mobility of Tryptic Peptides for High-Throughput CZE-MS Analysis. Anal. Chem. 2017, 89 (3), 2000–2008. 10.1021/acs.analchem.6b04544. 28208305 PMC5651133

[ref61] Chen D.; Lubeckyj R. A.; Yang Z.; McCool E. N.; Shen X.; Wang Q.; Xu T.; Sun L. Predicting Electrophoretic Mobility of Proteoforms for Large-Scale Top-Down Proteomics. Anal. Chem. 2020, 92 (5), 3503–3507. 10.1021/acs.analchem.9b05578. 32043875 PMC7543059

[ref62] Sanchez-Avila X.; Truong T.; Xie X.; Webber K. G. I.; Madisyn Johnston S.; Lin H. L.; Axtell N. B.; Puig-Sanvicens V.; Kelly R. T. Easy and Accessible Workflow for Label-Free Single-Cell Proteomics. J. Am. Soc. Mass Spectrom. 2023, 34 (10), 2374–2380. 10.1021/jasms.3c00240. 37594399 PMC11002963

[ref63] Specht H.; Emmott E.; Petelski A. A.; Gray Huffman R.; Perlman D. H.; Serra M.; Kharchenko P.; Koller A.; Slavov N. Single-cell proteomic and transcriptomic analysis of macrophage heterogeneity using SCoPE2. Genome Biol. 2021, 22 (1), 50 10.1186/s13059-021-02267-5. 33504367 PMC7839219

[ref64] Yang Z.; Zhang Z.; Chen D.; Xu T.; Wang Y.; Sun L. Nanoparticle-Aided Nanoreactor for Nanoproteomics. Anal. Chem. 2021, 93 (30), 10568–10576. 10.1021/acs.analchem.1c01704. 34297524 PMC9563093

[ref65] Perez-Riverol Y.; Bai J.; Bandla C.; Hewapathirana S.; García-Seisdedos D.; amatchinathan S.; Kundu D.; Prakash A.; Frericks-Zipper A.; Eisenacher M.; Walzer M.; Wang S.; Brazma A.; Vizcaíno J. A. The PRIDE database resources in 2022: a hub for mass spectrometry-based proteomics evidences. Nucleic Acids Res. 2022, 50 (D1), D543–D552. 10.1093/nar/gkab1038. 34723319 PMC8728295

